# Mitochondrial-derived gene expression in hibernation: tissue-specific responses in the thirteen-lined ground squirrel

**DOI:** 10.1098/rsob.240255

**Published:** 2025-08-13

**Authors:** Sarah Viola Emser, Eva Millesi

**Affiliations:** ^1^ Department of Behavioral and Cognitive Biology, University of Vienna, Vienna, Austria

**Keywords:** hibernation, transcriptome profiling, mitochondrial gene expression, mitochondrial-derived peptides, thirteen-lined ground squirrel

## Abstract

Hibernation is a remarkable physiological adaptation in many mammals, characterized by prolonged torpor and profound metabolic suppression. Despite its importance, the molecular mechanisms regulating mitochondrial-derived gene expression during hibernation remain poorly understood. In this study, we analysed mitochondrial gene expression across multiple tissues of the hibernating thirteen-lined ground squirrel (*Ictidomys tridecemlineatus*) using publicly available RNA sequencing (RNA-seq) data. We assessed all known mitochondrial DNA-derived transcripts—including mitochondrial mRNAs, mitochondrial-derived peptides and proteins (MDPs), rRNAs, and long non-coding RNAs (lncRNAs)—in the liver, adrenal gland, three brain regions, and brown adipose tissue (BAT) across different hibernation states.

Our findings reveal distinct tissue-specific expression patterns of mitochondrial transcripts. Differential expression was observed in three of the six tissues analysed (liver, adrenal gland, and BAT) while no significant changes were detected in the three brain regions. In tissues exhibiting differential expression, a consistent pattern emerged: lncRNAs such as Mdl1, Mdl1as, and lncCyb were generally upregulated, whereas mRNAs, including a novel transcript encoding the putative mitochondrial protein Rudel, were predominantly downregulated. These results provide new insights into mitochondrial gene regulation during hibernation and highlight tissue-specific adaptations at the level of mitochondrial gene expression.

**Figure:**
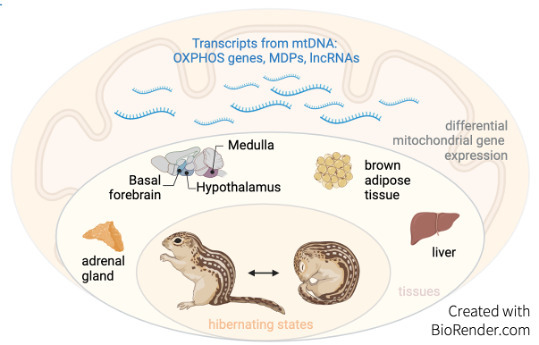

## 1. Introduction

Hibernation, a remarkable phenomenon observed in many mammals, is characterized by reduced metabolic rate and body temperature, alternating with short interbout arousals [1,2]. During deep torpor, body temperature drops to near ambient levels, often approaching the freezing point, while metabolic activity decreases to a fraction of the normal rate. This thermoregulatory adaptation is a highly efficient strategy to save energy, allowing animals to conserve resources and survive harsh environmental conditions and food scarcity [3]. In addition, other benefits such as reduced water loss, parasite load and even predation risk have been documented [4]. Hibernators usually stay in their burrows for five to seven months, relying on either body fat reserves accumulated before hibernation onset [5] or food caches in the burrow [6,7]. Periodically, hibernating animals undergo interbout arousals (IBAs), brief periods of euthermy, lasting for several hours, in which physiological functions are temporarily restored [1]. Despite spending extended periods in torpor, hibernating species exhibit remarkable resilience and do not suffer adverse effects from these extended periods of inactivity [8,9]. Instead, hibernation enables animals to survive harsh environmental conditions, conserve energy and endure prolonged periods of food scarcity, making it a crucial adaptation for survival in challenging environments.

At the molecular level, hibernation induces profound metabolic changes, particularly in energy metabolism pathways [10]. Central to these changes are mitochondria, organelles responsible for producing the majority of a cell’s energy through oxidative phosphorylation. Often referred to as the ‘powerhouses’ of the cell, mitochondria play a critical role in energy production by generating adenosine triphosphate (ATP) from nutrients. During hibernation, the function and regulation of mitochondrial activity become crucial, as the energy demands of the organism shift dramatically. Mitochondria must efficiently manage limited energy resources to support essential cellular functions while the organism remains in a state of torpor [11,12].

Mitochondria possess their own genome, separate from the nuclear genome, which encodes next to the classical 13 proteins involved in oxidative phosphorylation many other mitochondrial-derived peptides (MDPs), also termed microproteins, and several classes of non-coding RNA. MDPs are a constantly growing group derived from both strands of mitochondrial DNA (mtDNA), and consist currently of Humanin [13], MOTS-c [14], SHLP1-6 [15], SHMOOSE [16], MTALTND4 [17], Gau [18] and CYTB-187AA [19]. Next to protein-coding genes, mtDNA encodes two rRNAs and 22 tRNAs that participate in mitochondrial translation, and the class of mitochondria-encoded circRNAs (mecciRNAs), mitochondria-encoded double-stranded RNAs (mt-dsRNAs) [20] and long non-coding RNAs (lncRNAs), namely, long intergenic non-coding RNA predicting cardiac remodelling (LIPCAR), sense non-coding mtRNA (SncmtRNA), antisense non-coding mtRNA1 and 2 (ASncmtRNA1 and 2), 7SRNA, mitochondrial D-loop 1 (MDL1), mitochondrial D-loop 1 antisense (MDL1AS), lncND5, lncND6 and lncCYB [21].

Despite the importance of mitochondrial gene expression in regulating mitochondrial function, comprehensive analyses of mitochondrial gene expression during hibernation are lacking, particularly at the transcriptomic level. Numerous studies have investigated gene expression changes in hibernating animals using RNA-seq analysis [22–28], yet genes that derive from mtDNA are frequently overlooked or omitted from these analyses [25,29]. Standard RNA-seq data analysis typically maps the reads to the reference genome or transcriptome that does not include mitochondrial-derived transcripts for non-model species. This omission represents a significant gap in our understanding of the molecular mechanisms underlying metabolic adaptations during hibernation, given that up to 40% of RNA reads align to the mitochondrial genome [27]. The research groups led by M. T. Andrews and S. Martin have conducted extensive gene expression studies on hibernation, the former developed a transcriptome browser for *Ictidomys tridecemlineatus* (https://d.umn.edu/∼mhampton/GB18.html), which includes information on 14 261 genes but not mitochondrial genes. Mitochondrial-targeted genes that refer to nuclear-encoded genes producing proteins or RNAs directed to the mitochondria are often identified as differentially expressed during hibernation [27,30]. These genes are different from ‘mitochondrial-derived genes’, which are encoded by the mitochondrial genome itself. The former are transcribed in the nucleus and then transported to the mitochondria, while the latter are transcribed and translated within mitochondria. Microarray studies that did consider the latter form of mitochondrial transcripts found differential expression of *mt-Co1* and *mt-Atp6/8* [31], as well as *mt-Cyb*, *mt-Co2* and *mt-Nd1* during hibernation [28]. Previous work also identified differential expression of the MDP SHLP6 in brown adipose tissue (BAT) of the thirteen-lined ground squirrel [32], prompting further investigation into MDP expression during hibernation.

This study conducted a comprehensive analysis of mitochondrial gene expression to enhance understanding of transcriptional dynamics and the role of mitochondrial gene expression in hibernation physiology as well as of the molecular basis of metabolic suppression and energy conservation in hibernating animals. Utilizing an up-to-date mitochondrial transcriptome, we examined mitochondrial mRNAs, rRNAs, lncRNAs and MDP-encoding RNAs using publicly available RNA-seq data produced for diverse tissues of the hibernator *I. tridecemlineatus*. By investigating the expression profiles of mitochondrial genes during states of hibernation, we sought to elucidate the transcriptional regulation of mitochondrial function and to identify potential molecular pathways involved in metabolic adaptations during hibernation.

## 2. Material and methods

### 2.1. Database search for RNA-seq data of the thirteen-lined ground squirrel

Transcriptome data publicly available at the Gene Expression Omnibus (GEO) and Sequence Read Archive (SRA) repositories of NCBI were screened for hibernating species such as *I. tridecemlineatus* (thirteen-lined ground squirrel). Sequencing protocols that did not contain mitochondrial fractions (like single-cell nucleus RNA-seq), small or miRNA-seq and high-throughput sequencing platforms other than Illumina were excluded. Inclusion criteria comprised at least two collection time points (one in the active period and the other during hibernation), tissue from native animals (not treated after dissection, immediately sequenced) that were in their natural seasonal state (no artificial hibernation deprivation), and at least three biological replicates per collection time point. The datasets used in this study are summarized in electronic supplementary material, table S1, including their metadata concerning tissue, read length, replicates, accession numbers and references.

### 2.2. Reference mitochondrial transcriptome for the thirteen-lined ground squirrel

Gene features of the complete mitochondrial reference genome from the thirteen-lined ground squirrel were downloaded from NCBI (NC_027278.1) and combined with open reading frames (ORFs) of putative MDP and non-coding RNAs. In detail, MDPs were predicted based on homology of ORFs to human MDP sequences, namely, MOTS-c, (s)Humanin, SHLP1-6, Gau, MTALTND4, SHMOOSE, CYTB-187AA and putative ORF that was depicted in [17], and that we termed Rudel (Russian doll of electron carrier and energy transducer ATPase genes) according to its antisense location of MT-ATP6/8 (electronic supplementary material). The non-coding RNAs 7SRNA, LIPCAR, lncCYB, lncND5, lncND6, MDL1, MLD1AS, SncmtRNA, ASncmtRNA1 and 2 were similarly predicted. The transcriptome file is available in Data sheet 1. Transcripts lacking polyadenylation, such as mt-tRNAs, which were incompatible with the poly-A sequencing library, were excluded.

### 2.3. RNA-seq data analysis

For each dataset, raw RNA-seq reads were downloaded from NCBI to the Galaxy server [33] (usegalaxy.org). Transcript abundance was quantified using Kallisto quant v. 0.46.2 [34]. For strand-specific libraries (in the case of liver and brain regions), the option for strand-specific reads was set to first read reverse. Variance-mean dependence of count data and their differential abundance based on negative binomial distribution were estimated by the DESeq2 software [35]. A filtering step was included, retaining only those genes in each tissue where *rlog* was ≥ 7 in all samples. Differentially expressed genes were defined as those with an adjusted *p*‐value (*p*_adj_) ≤ 0.05, based on the likelihood ratio test across all states, with the model accounting for the effect of sex as a covariate.

## 3. Results

To evaluate tissue-specific mitochondrial transcriptome profiling for hibernation, we screened the SRA database of NCBI for transcriptomic datasets of hibernators. Most transcriptome studies on hibernation that matched our inclusion and exclusion criteria were conducted using tissues of the thirteen-lined ground squirrel. We therefore focused our mitochondrial transcript expression analysis on this hibernator model. RNA-seq data of the following tissues matched our study criteria: adrenal gland, BAT, three brain regions, namely, medulla, hypothalamus and forebrain, and liver (electronic supplementary material, table S1). Mitochondrial percentage of total reads ranged between 41.1 ± 4.2% (BAT) and 0.3 ± 0.1% (adrenal gland). The latter percentage differed significantly from the value reported in the original study on the adrenal gland (4.7 ± 1.7 [22]). Selected datasets were constructed using poly(A) selection, and either sequenced from stranded libraries (liver and brain regions) or single-ended in the case of adrenal gland and BAT. Strand-specific reads are particularly important for the mitochondrial genome, as both strands are transcribed and encode biologically relevant transcripts. This enabled us to distinguish between sense and antisense expression, for example, between *mt-Atp6/8* and *Rudel*, *mt-Co1* and *Gau*, or Mdl1 and Mdl1as.

**Figure 1.**
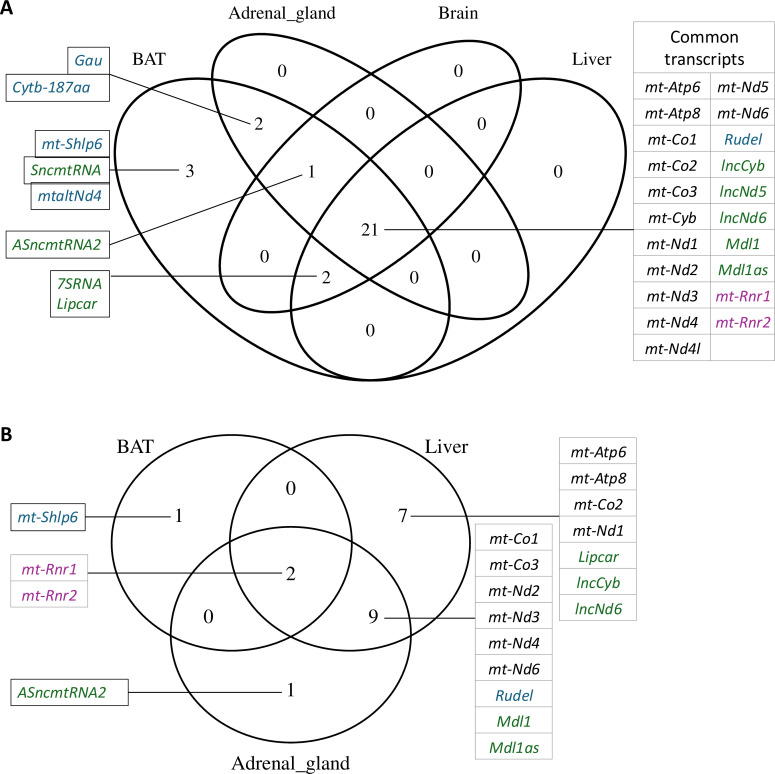
Venn diagram of detected (A) and differentially expressed (B) mitochondrial transcripts in the six tissues. Between 24 and 29 (63−76%) transcripts were detected in the different tissues after filtering the data with *rlog* > 7. Of these, 21 transcripts (75%) were common to all six tissues. For clarity, the three brain regions were grouped together, as no difference in transcript detection was observed among them. Also, none of the brain regions exhibited significant differential mitochondrial gene expression. Classical mRNAs are shown in black, MDP transcripts in blue, rRNAs in purple and lncRNAs in green.

Reads of the selected datasets were aligned to a mitochondrial-derived transcriptome of the thirteen-lined ground squirrel which includes all known peptide- and protein-coding mitochondrial genes—including MDPs—as well as non-coding RNAs such as the two rRNAs and several lncRNAs, totaling 38 transcripts (see §2 for details, Data sheet 1). After a filtering step, we confidently identified 29 transcripts across all tissues. Of those, 21 transcripts (75%) were commonly expressed in all analysed tissues (figure 1A). They consisted of the 13 classical protein-coding genes, the two rRNAs, five lncRNAs and a previously undescribed novel mitochondrial-derived gene, *Rudel* (electronic supplementary material). Tissue-specific expression was observed in the case of the MDPs Gau and Cytb-187aa (in adrenal gland and BAT), mt-Shlp6, SncmtRNA and mtaltNd4 (BAT) and the lncRNAs ASncmtRNA2 (BAT, brain and adrenal gland), LIPCAR and 7RNA (all but adrenal gland; figure 1). Ten of the generated transcripts were not detected in any of the tissues, namely, MOTS-c, SHLP1–5, Humanin and SHMOOSE from the class of MDP-coding RNAs, and SncmtRNA and ASncmtRNA1 from the class of lncRNAs. Except for Humanin [36], whose transcript may be too short for detection in our setting, none of the undetected transcripts have been observed previously in the thirteen-lined ground squirrel.

### 3.1. Differential expression of mitochondrial transcripts during hibernation

**Figure 2.**
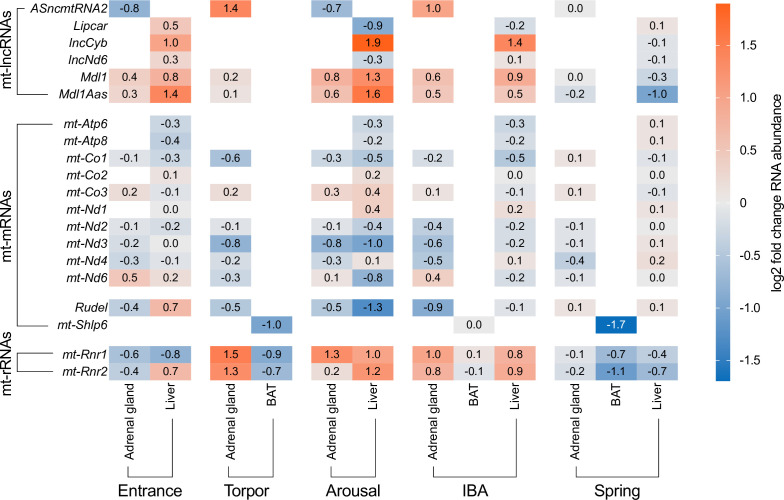
Significantly up- and downregulated mitochondrial transcripts in the three tissues that showed differential expression across hibernation states (entrance, torpor, arousal, interbout arousal (IBA), and spring), compared to the summer active state, in the thirteen-lined ground squirrel (see electronic supplementary material, tables S2–S7). Columns are grouped according to hibernation states and tissue, while rows correspond to individual mitochondrial transcripts. The colour gradient ranges from blue (downregulation) to orange (upregulation), with white indicating no significant change (*p_adj_* > 0.05) in transcript expression. Notable trends include the upregulation of lncRNAs and rRNAs, and the downregulation of mRNAs.

To investigate up- or downregulation of mitochondrial transcripts during hibernation, we compared their abundance across common hibernation states—entrance, torpor, arousal, IBA and spring—using the software package DESeq2 and the summer active state (October) as a reference (electronic supplementary material, tables S2–S7). Out of the 29 detected transcripts, 18 (62%) were differentially expressed (*p*_adj_ ≤ 0.05) in a tissue-specific manner (figure 1B). Notably, expression of mitochondrial transcripts was unchanged in the three examined brain regions. In contrast, 18 transcripts were differentially expressed in the liver, 16 in the adrenal gland and three in BAT (figure 2).

Opposite expression patterns were observed between non-coding RNAs (ncRNAs) and coding RNAs during hibernation. While coding RNAs showed significant downregulation, ncRNAs including lncCyb, Mdl1 and Mdl1as were consistently upregulated. Notably, mRNAs coding for the mitochondrial subunits of the ATPase complex (*Atp6/8*) and for *mt-Co1*, *mt-Nd2* and *mt-Nd3* exhibited consistent downregulation during hibernation including IBA. These findings suggest distinct regulatory roles for ncRNAs and coding RNAs in modulating mitochondrial function during hibernation. The importance of SHLP6 in BAT was described previously [32]. Here, we confirmed the reported expression pattern using a slightly different workflow (figure 2).

## 4. Discussion

This study underscores the critical role of mitochondrial-derived transcripts in hibernation. Differential expression of many mitochondrial transcripts across various tissues highlights their importance in adapting to the unique physiological demands of hibernation. Inclusion of mitochondrial transcripts in transcriptome analyses is essential for a comprehensive understanding of the molecular mechanisms underlying this process.

Our findings revealed tissue-specific regulation of MDPs, suggesting that distinct mitochondrial pathways are engaged in different tissues to facilitate hibernation. Notably, BAT, adrenal gland and liver exhibited significant transcriptional changes, while mitochondrial genes were not differentially expressed in the three brain regions analysed. The absence of alteration in medulla, forebrain and hypothalamus is intriguing. The stability may reflect the brain’s stringent metabolic requirements, prioritizing energy consistency over flexibility during hibernation. Unlike peripheral tissues, which undergo significant metabolic reprogramming, the brain likely employs alternative mechanisms—such as modifications on the post-transcriptional and/or the protein level—to maintain critical functions.

Future studies should explore their specific nature such as phosphorylation or acetylation, and assess mitochondrial function at the proteomic and metabolomic levels to uncover how the brain supports hibernation-specific adaptation.

We observed a general trend of mRNA downregulation and lncRNA upregulation across the hibernation states of entrance, torpor, arousal and IBA. The downregulation of *mt-Shlp6* may be linked to its role in promoting apoptosis. While some cells undergo apoptosis during the transition to torpor, the majority of cells must be safeguarded to prevent extensive cell loss.

Previous studies have demonstrated differential expression of specific mitochondrial transcripts during hibernation. Hittel and Storey were the first to identify changes in the expression of *mt-Co1* and *mt-Atp6/8* in the kidneys of the thirteen-lined ground squirrel [31]. While they investigated potential nuclear co-regulation of subunits for the oxidative phosphorylation system, they did not find such regulation for the nuclear genes encoding Cox4 or ATP synthase a.

Similarly, another study [28] reported differential expression of the mitochondrial genes *mt-Cyb*, *mt-Co2* and *mt-Nd1* in the heart. However, the study likewise did not observe co-regulation with nuclear-encoded mitochondrial-targeted genes. These findings suggest that capturing co-regulation between mitochondrial and nuclear genes is complex and may involve more intricate mechanisms [37].

Our current analysis confirms and expands upon these earlier findings by providing a more comprehensive view of mitochondrial transcript regulation. Differential expression of *mt-Co1* was observed in adrenal gland and liver. The gene expression changes of *mt-Atp6/8* were also confirmed in our analysis, with a downregulation in liver during all states of hibernation. The other complex subunits with observed up- and downregulations did not present such a clear pattern. It remains unclear whether the differential expression leads to varying protein abundance or if the translatome is subjected to an unknown regulator.

One notable finding was the influence of read length and library settings on the detection of MDP-encoding RNAs. Given that some MDPs are derived from small ORFs, such as MOTS-c, which has an ORF length of only 36 nucleotides and an unknown transcript length, the choice of sequencing parameters can significantly affect the ability to detect transcripts of these peptides. This highlights the need to carefully consider the sequencing strategy in future studies to ensure accurate detection and quantification of transcripts encoding small mitochondrial peptides.

Particularly intriguing was the consistent tissue expression of the novel putative ORF encoding Rudel. It exhibits a length of 663 nucleotides and is located on the antisense strand of *mt-Atp6/8*. Detection benefited from the use of strand-specific libraries providing higher sequence confidence. Its significant downregulation except for the *entrance* state in liver suggests an important role for this transcript in hibernation physiology. Further studies are needed to elucidate the function of the putative ORF and its potential contributions to metabolic adaptations during hibernation.

Our analysis did not detect the lncRNAs SncmtRNA and ASncmtRNA1, both previously described for mice. Because their lengths vary significantly across species, we used for analysis the lengths reported for the closely related mouse. Interestingly, we identified ASncmtRNA2 but not SncmtRNA or ASncmtRNA1, suggesting that the lengths of these lncRNAs in the thirteen-lined ground squirrel may differ from those in mice and could be more similar to their human counterparts. This finding highlights the need for further investigation to determine the precise lengths and functional roles of these lncRNAs in hibernating animals.

The tissue-specific expression patterns observed for MDPs, such as Gau, SHLP6, MTALTND4 and CYTB-187AA, as well as for the lncRNAs ASncmtRNA2, LIPCAR and 7RNA, further emphasized the complexity of mitochondrial regulation during hibernation.

The significant deregulation of 18 out of 29 detected transcripts in liver highlights the dynamic nature of mitochondrial gene expression during hibernation and suggests specific mitochondrial pathways that are crucial for different phases of hibernation.

Future research should continue to explore the functions of newly identified mitochondrial transcripts and further investigate the tissue-specific regulation of mitochondrial genes to fully elucidate the mechanisms that enable hibernating animals to survive prolonged periods of metabolic suppression and food scarcity.

## 5. Conclusions

This descriptive study provides a comprehensive analysis of mitochondrial gene expression in hibernating animals, highlighting the significant role of mitochondrial-derived transcripts and the tissue-specific regulation of mitochondrial functions during hibernation. Our findings demonstrate the importance of including mitochondrial transcripts in transcriptomic analyses to capture important molecular adaptations that occur during hibernation. The identification of novel transcripts, such as the putative mitochondrial gene *Rudel*, and the differential expression of MDP-encoding RNAs and lncRNAs, offer new insights into the mitochondrial expression patterns during hibernation. Future research should continue to investigate these mitochondrial transcripts and their roles in hibernation physiology. This can enhance our understanding of the complex interplay between mitochondrial function and the adaptive strategies of hibernating species.

## Data Availability

The datasets used in this study are summarized in electronic supplementary material, table 1, including their metadata concerning tissue, read length, replicates, accession numbers and references. Electronic supplementary material is available online [38].

## References

[1] Ruf T, Geiser F. 2015 Daily torpor and hibernation in birds and mammals. Biol. Rev. Camb. Philos. Soc. **90**, 891–926. (10.1111/brv.12137) 25123049 PMC4351926

[2] Buck CL, Barnes BM. 2000 Effects of ambient temperature on metabolic rate, respiratory quotient, and torpor in an Arctic hibernator. Am. J. Physiol. Regul. Integr. Comp. Physiol. **279**, R255–62. (10.1152/ajpregu.2000.279.1.R255) 10896889

[3] Mohr SM, Bagriantsev SN, Gracheva EO. 2020 Cellular, molecular, and physiological adaptations of hibernation: the solution to environmental challenges. Annu. Rev. Cell Dev. Biol. **36**, 315–338. (10.1146/annurev-cellbio-012820-095945) 32897760

[4] Geiser F, Brigham RM. 2012 The other functions of torpor. In Living in a seasonal world: thermoregulatory and metabolic adaptations (eds R Thomas, B Claudia, A Walter, M Eva), pp. 109–121. Berlin, Germany: Springer. (10.1007/978-3-642-28678-0_10)

[5] Sheriff MJ, Fridinger RW, Tøien øivind, Barnes BM, Buck CL. 2013 Metabolic rate and prehibernation fattening in free-living arctic ground squirrels. Physiol. Biochem. Zool. **86**, 515–527. (10.1086/673092) 23995482

[6] Humphries MM, Thomas DW, Kramer DL. 2003 The role of energy availability in mammalian hibernation: a cost‐benefit approach. Physiol. Biochem. Zool. **76**, 165–179. (10.1086/367950) 12794670

[7] Siutz C, Franceschini C, Millesi E. 2016 Sex and age differences in hibernation patterns of common hamsters: adult females hibernate for shorter periods than males. J. Comp. Physiol. B Biochem. Syst. Environ. Physiol. **186**, 801–811. (10.1007/s00360-016-0995-z) PMC4933728 27138337

[8] Miyazaki M, Shimozuru M, Tsubota T. 2019 Skeletal muscles of hibernating black bears show minimal atrophy and phenotype shifting despite prolonged physical inactivity and starvation. PLoS One **14**, e0215489. (10.1371/journal.pone.0215489) 30998788 PMC6472773

[9] De Vrij EL, Bouma HR, Henning RH, Cooper ST. 2023 Hibernation and hemostasis. Front. Physiol. **14**, 1207003. (10.3389/fphys.2023.1207003) 37435313 PMC10331295

[10] Andrews MT. 2019 Molecular interactions underpinning the phenotype of hibernation in mammals. J. Exp. Biol. **222**, jeb160606. (10.1242/jeb.160606) 30683731

[11] Ballinger MA, Schwartz C, Andrews MT. 2017 Enhanced oxidative capacity of ground squirrel brain mitochondria during hibernation. Am. J. Physiol. Regul. Integr. Comp. Physiol. **312**, R301–R310. (10.1152/ajpregu.00314.2016) 28077389 PMC5402005

[12] Ballinger MA, Hess C, Napolitano MW, Bjork JA, Andrews MT. 2016 Seasonal changes in brown adipose tissue mitochondria in a mammalian hibernator: from gene expression to function. Am. J. Physiol. Regul. Integr. Comp. Physiol. **311**, R325–R336. (10.1152/ajpregu.00463.2015) 27225952

[13] Hashimoto Y *et al*. 2001 A rescue factor abolishing neuronal cell death by a wide spectrum of familial Alzheimer’s disease genes and Abeta. Proc. Natl Acad. Sci. USA **98**, 6336–6341. (10.1073/pnas.101133498) 11371646 PMC33469

[14] Lee C *et al*. 2015 The mitochondrial-derived peptide MOTS-c promotes metabolic homeostasis and reduces obesity and insulin resistance. Cell Metab. **21**, 443–454. (10.1016/j.cmet.2015.02.009) 25738459 PMC4350682

[15] Cobb LJ *et al*. 2016 Naturally occurring mitochondrial-derived peptides are age-dependent regulators of apoptosis, insulin sensitivity, and inflammatory markers. Aging **8**, 1–14. (10.18632/aging.100943) 27070352 PMC4925829

[16] Miller B *et al*. 2023 Correction: Mitochondrial DNA variation in Alzheimer’s disease reveals a unique microprotein called SHMOOSE. Mol. Psychiatry **28**, 1827. (10.1038/s41380-023-01956-w) 36658336

[17] Kienzle L *et al*. 2023 A small protein coded within the mitochondrial canonical gene nd4 regulates mitochondrial bioenergetics. BMC Biol. **21**, 111. (10.1186/s12915-023-01609-y) 37198654 PMC10193809

[18] Faure E, Delaye L, Tribolo S, Levasseur A, Seligmann H, Barthélémy RM. 2011 Probable presence of a ubiquitous cryptic mitochondrial gene on the antisense strand of the cytochrome oxidase I gene. Biol. Direct **6**, 56. (10.1186/1745-6150-6-56) 22024028 PMC3214167

[19] Hu Z *et al*. 2024 A novel protein CYTB-187AA encoded by the mitochondrial gene CYTB modulates mammalian early development. Cell Metab. **36**, 1586–1597. (10.1016/j.cmet.2024.04.012) 38703762

[20] Wiatrek DM, Candela ME, Sedmík J, Oppelt J, Keegan LP, O’Connell MA. 2019 Activation of innate immunity by mitochondrial dsRNA in mouse cells lacking p53 protein. RNA **25**, 713–726. (10.1261/rna.069625.118) 30894411 PMC6521600

[21] Ren B, Guan MX, Zhou T, Cai X, Shan G. 2023 Emerging functions of mitochondria-encoded noncoding RNAs. Trends Genet. **39**, 125–139. (10.1016/j.tig.2022.08.004) 36137834

[22] Gillen AE, Epperson LE, Orlicky DJ, Fu R, Martin SL. 2023 Adrenal gene expression dynamics support hibernation in 13-lined ground squirrels. Physiol. Genom. **55**, 155–167. (10.1152/physiolgenomics.00162.2022) 36847440

[23] Gillen AE, Fu R, Riemondy KA, Jager J, Fang B, Lazar MA, Martin SL. 2021 Liver transcriptome dynamics during hibernation are shaped by a shifting balance between transcription and RNA stability. Front. Physiol. **12**, 662132. (10.3389/fphys.2021.662132) 34093224 PMC8176218

[24] Fu R, Gillen AE, Grabek KR, Riemondy KA, Epperson LE, Bustamante CD, Hesselberth JR, Martin SL. 2020 Dynamic RNA regulation in the brain underlies physiological plasticity in a hibernating mammal. Front. Physiol. **11**, 624677. (10.3389/fphys.2020.624677) 33536943 PMC7848201

[25] Vermillion KL, Anderson KJ, Hampton M, Andrews MT. 2015 Gene expression changes controlling distinct adaptations in the heart and skeletal muscle of a hibernating mammal. Physiol. Genom. **47**, 58–74. (10.1152/physiolgenomics.00108.2014) PMC4346737 25572546

[26] Schwartz C, Hampton M, Andrews MT. 2013 Seasonal and regional differences in gene expression in the brain of a hibernating mammal. PLoS One **8**, e58427. (10.1371/journal.pone.0058427) 23526982 PMC3603966

[27] Hampton M, Melvin RG, Andrews MT. 2013 Transcriptomic analysis of brown adipose tissue across the physiological extremes of natural hibernation. PLoS One **8**, e85157. (10.1371/journal.pone.0085157) 24386461 PMC3875542

[28] Hampton M, Melvin RG, Kendall AH, Kirkpatrick BR, Peterson N, Andrews MT. 2011 Deep sequencing the transcriptome reveals seasonal adaptive mechanisms in a hibernating mammal. PLoS One **6**, e27021. (10.1371/journal.pone.0027021) 22046435 PMC3203946

[29] Goropashnaya AV, Barnes BM, Fedorov VB. 2020 Transcriptional changes in muscle of hibernating Arctic ground squirrels (Urocitellus parryii): implications for attenuation of disuse muscle atrophy. Sci. Rep. **10**, 9010. (10.1038/s41598-020-66030-9) 32488149 PMC7265340

[30] Luan Y, Ou J, Kunze VP, Qiao F, Wang Y, Wei L, Li W, Xie Z. 2018 Integrated transcriptomic and metabolomic analysis reveals adaptive changes of hibernating retinas. J. Cell. Physiol. **233**, 1434–1445. (10.1002/jcp.26030) 28542832

[31] Hittel DS, Storey KB. 2002 Differential expression of mitochondria-encoded genes in a hibernating mammal. J. Exp. Biol. **205**, 1625–1631. (10.1242/jeb.205.11.1625) 12000807

[32] Emser SV, Spielvogel CP, Millesi E, Steinborn R. 2023 Mitochondrial polymorphism m.3017C>T of SHLP6 relates to heterothermy. Front. Physiol. **14**, 1207620. (10.3389/fphys.2023.1207620) 37675281 PMC10478271

[33] The Galaxy Community. 2024 The Galaxy platform for accessible, reproducible, and collaborative data analyses: 2024 update. Nucleic Acids Res. **52**, W83–W94. (10.1093/nar/gkae410) 38769056 PMC11223835

[34] Bray NL, Pimentel H, Melsted P, Pachter L. 2016 Near-optimal probabilistic RNA-seq quantification. Nat. Biotechnol. **34**, 525–527. (10.1038/nbt.3519) 27043002

[35] Love MI, Huber W, Anders S. 2014 Moderated estimation of fold change and dispersion for RNA-seq data with DESeq2. Genome Biol. **15**, 550. (10.1186/s13059-014-0550-8) 25516281 PMC4302049

[36] Szereszewski KE, Storey KB. 2019 Identification of a prosurvival neuroprotective mitochondrial peptide in a mammalian hibernator. Cell Biochem. Funct. **37**, 494–503. (10.1002/cbf.3422) 31387137

[37] Soto I, Couvillion M, Hansen KG, McShane E, Moran JC, Barrientos A, Churchman LS. 2022 Balanced mitochondrial and cytosolic translatomes underlie the biogenesis of human respiratory complexes. Genome Biol. **23**, 170. (10.1186/s13059-022-02732-9) 35945592 PMC9361522

[38] Emser SV, Millesi E. 2025 Supplementary material from: Mitochondrial-Derived Gene Expression in Hibernation: Tissue-Specific Responses in the Thirteen-Lined Ground Squirrel. Figshare. (10.6084/m9.figshare.c.7945359.v1)40795995

